# New Appliances & Things Medical

**Published:** 1911-02-11

**Authors:** 


### NEW APPLIANCES & THINGS MEDICAL.

DIAMALT (THE BRITISH DIAMALT CO., 11 TO 13, SOUTHWARK STREET, S.E.).

We have received two specimens of diamalt, which is a registered name for an extract of malt of high diastatic power. One consists of diamalt itself, the other of diamalt combined with 33 per cent. of cod-liver oil. We have every reason to believe that both the malt and the cod-liver oil used for these preparations are of the best quality and free from impurity. Malt extracts are generally prescribed for the purpose of reinforcing a given diet, especially in carbo-hydrates, and also for assisting by virtue of the diastase they contain in the actual digestion of starch. Some patients appear to experience considerable difficulty in converting starch into sugar. Theoretically an actively diastatic malt-extract should facilitate this process, and we find that it actually does so in practice. The extract before us is one of the most active, if not the most active, extract on the market, although malt-extracts as reinforcers of the diet are for what they actually yield relatively expensive, yet, nevertheless, as a fact it is found that maltose is well borne by patients and is superior to other cheaper forms of carbo-hydrate. We think the preparations before us are reliable and to be recommended.

